# Disparity of ophthalmic surgeries in Japan

**DOI:** 10.1371/journal.pone.0347587

**Published:** 2026-04-24

**Authors:** Teruna Shiga, Ayane Tanaka, Yoshiko Fukuda, Kenji Kashiwagi

**Affiliations:** Department of Ophthalmology, Faculty of Medicine, University of Yamanashi, Yamanashi, Japan; Shinshu University School of Medicine, JAPAN

## Abstract

**Purpose:**

Although regional disparities in access to surgical care have been reported across medical specialties, nationwide evaluations of variation in ophthalmic surgical services remain limited. This study examined prefecture-level differences in major ophthalmic surgeries in Japan using publicly available claims data.

**Methods:**

We analyzed the ninth release of the National Database of Health Insurance Claims and Specific Health Checkups of Japan (NDB Open Data) for fiscal year 2022 (April 2022–March 2023). Prefecture-level procedure counts were extracted for cataract surgery (K282), vitrectomy (K279–K281 and related codes), glaucoma surgery (relevant K-codes), and corneal transplantation. To reduce potential underestimation in non–Diagnosis Procedure Combination settings, ophthalmology-related procedures reimbursed under the Short-Stay Surgery Basic Fee 3 (A400) were identified and incorporated according to predefined mapping rules. Surgical rates per 100,000 population and per board-certified ophthalmologist were calculated. Regional inequality was quantified using population-weighted Gini coefficients. Associations between ophthalmologist density and surgical volume were assessed using Pearson’s correlation coefficients.

**Results:**

Nationwide totals were 1,777,502 cataract surgeries, 154,336 vitrectomies, 80,753 glaucoma surgeries, and 2,895 corneal transplantations. Prefecture-level surgical rates per 100,000 population varied 1.8-fold for cataract surgery, 3.3-fold for vitrectomy, and 7.8-fold for glaucoma surgery. Several prefectures reported no corneal transplantation cases. Population-weighted Gini coefficients were 0.064 for cataract surgery, 0.125 for vitrectomy, 0.190 for glaucoma surgery, and 0.351 for corneal transplantation. Similar patterns were observed after adjusting for age structure, indicating that regional disparities were not solely explained by demographic differences. Ophthalmologist density varied 2.5-fold across prefectures and was positively correlated with surgical volume for cataract surgery and vitrectomy, but not for glaucoma surgery.

**Conclusions:**

Using nationwide claims data, we identified substantial regional variation in major ophthalmic surgical procedures in Japan, with greater inequality observed for more specialized surgeries. These findings provide a population-level description of ophthalmic surgical distribution and may inform future evaluations of healthcare resource allocation.

## Introduction

Major ophthalmic diseases, including cataracts, glaucoma, and several retinal disorders, are associated with aging, which is a major risk factor for disease onset. Accordingly, with the rapid aging of the global population, the prevalence of these conditions has continued to increase, which has been accompanied by a reported increase in the number of ophthalmic surgical procedures [1–5]. In contrast, the uneven geographic distribution of medical specialists, including ophthalmologists, as well as medical facilities, has been documented across many medical disciplines [6–10]. Such maldistribution of healthcare professionals may hinder patients’ access to medical services and limit their ability to receive appropriate and timely treatment.

Surgical intervention is a central component of ophthalmic care and plays a crucial role in preserving and improving vision. However, because of the uneven distribution of ophthalmologists, some patients may be unable to undergo surgical treatment at an appropriate time, which may result in irreversible visual impairment. Despite these concerns, few studies in Japan have systematically evaluated the impact of regional disparities in the provision of ophthalmic surgical care.

In the present study, we investigated the real-world status of major ophthalmic surgical procedures in Japan, focusing on cataract surgery as a fundamental ophthalmic intervention, as well as vitreoretinal surgery, corneal transplantation, and glaucoma surgery, which require higher levels of surgical expertise. This analysis was primarily based on registration data from the National Database of Health Insurance Claims and Specific Health Checkups of Japan (NDB) Open Data (referred to as NDB Open Data), provided by the Ministry of Health, Labour and Welfare [11].

## Methods

### Data use and ethical considerations

All the data included in the NDB Open Data are fully anonymized, and therefore ethical approval was not needed. Furthermore, the use of these data does not require application to or permission from the Ministry of Health, Labour and Welfare, the authority responsible for managing the database.

### Data collection and definitions

Data were extracted from the 9th release of the National Database of Health Insurance Claims and Specific Health Checkups of Japan, which aggregates claims from April 2022 to March 2023, as shown in Table 1. Surgical procedures were identified from the “surgery (K)” category of the Japanese medical fee schedule and were limited to those in Chapter 4 (Ophthalmology). Both outpatient and inpatient procedures were included. The specific procedure codes analyzed are listed in Table 1. Cataract surgery was identified using the procedure code K282. Vitreoretinal and glaucoma surgeries were classified into multiple groups for analysis according to the surgical complexity, as detailed below.

**Table 1:** List of surgical procedures included in the analysis.

Major category	Surgical procedure code	Procedure code	1. Procedure name
Cataract surgery	K-282, (Inc, A400)	150253010	2. Lens reconstruction surgery with intraocular lens implantation (other types)
		150315610	3. Lens reconstruction surgery without intraocular lens implantation
		150356210	4. Lens reconstruction surgery with sutured intraocular lens implantation
	Short-Stay Surgery Basic Fee 3 (A400)	190179210	5. Lens reconstruction surgery 1: with intraocular lens implantation, other types, unilateral
		190195910	6. Lens reconstruction surgery 1: with intraocular lens implantation, other types, bilateral
		190269610	7. Lens reconstruction surgery 2: without intraocular lens implantation, unilateral
		190269810	8. Lens reconstruction surgery 2: without intraocular lens implantation, bilateral
Vitreoretinal surgery	K-277–2	150291810	9. Submacular surgery
	K-279	150090410	10. Vitrectomy
	K-280	150090610	11. Microscopic vitrectomy via pars plana (other procedures)
	K-280–2	150356110	12. Vitrectomy including retinal attachment tissues using an intraocular endoscope
	K-281	150252810	13. Surgery for proliferative vitreoretinopathy
Glaucoma surgery	K-268 (Inc, A400)	150335910	14. Glaucoma surgery (filtering surgery)
		150356010	15. Glaucoma surgery with implantation of a glaucoma drainage device (without plate)
		150373010	16. Glaucoma surgery with implantation of a glaucoma drainage device (with plate)
		150395150	17. Combined intraocular drainage surgery with lens reconstruction
		150435810	18. Glaucoma outflow pathway reconstruction (ab interno approach)
		150427210	19. Glaucoma outflow pathway reconstruction (other procedures)
	Short-Stay Surgery Basic Fee 3 (A400)	190269410	20. Glaucoma surgery 6: combined intraocular drainage surgery with lens reconstruction
		190269510	21. Glaucoma surgery 6: combined intraocular drainage surgery with lens reconstruction (for patients receiving long-term care)
Corneal transplantation	K-259	150086210	22. Corneal transplantation

### Vitreoretinal surgery

Vitreoretinal surgeries were analyzed using all the procedures listed in Table 1 and were further classified into three groups:

Group 1: Vitrectomy (K279);

Group 2: Microscopic vitrectomy via the pars plana (K280; including procedures with other indications and those involving retinal attachment tissues);

Group 3: Surgery for proliferative vitreoretinopathy (K281), vitrectomy involving retinal attachment tissues performed with an intraocular endoscope (K280-2), and submacular surgery (K277-2).

### Glaucoma surgery

All glaucoma-related procedures listed in Table 1 were included and further categorized into two groups:

Group 1: Glaucoma surgery (filtering surgery) and glaucoma surgery involving implantation of a glaucoma drainage device with a plate.

Group 2 Combined intraocular drainage surgery with lens reconstruction, glaucoma surgery involving implantation of a glaucoma drainage device without a plate, glaucoma outflow pathway reconstruction (other procedures), and ab interno outflow pathway reconstruction.

### Population and ophthalmologist data

Prefectural population data were obtained from the population estimates as of October 1, 2024, published by the Ministry of Health, Labour and Welfare [12]. Data on the number of board-certified ophthalmologists by prefecture were obtained from publicly available statistics released by the Japanese Ophthalmological Society [13].

### Adjustment for short-stay surgery (A400)

In non–diagnosis procedure combination (non-DPC) settings, some inpatient surgical cases are reimbursed under the Short-Stay Surgery Basic Fee 3 (A400), in which perioperative management and selected procedures are bundled and recorded under A400-related procedure codes rather than individual surgical K-codes. To minimize the underestimation of surgical volume, we additionally identified ophthalmology-related A400 (“short-stay”) procedure codes in the NDB Open Data for FY2022 and added their counts to the corresponding surgical categories.

This approach follows NDB Open Data guidance and indicates that procedure counts may shift between A400 and K-code tables across fiscal years because of changes in reimbursement rules and that combining both sources reduces artificial year-to-year fluctuations. Counts of A400-related procedures were aggregated at the prefectural level and were consistently integrated across all 47 prefectures to ensure comparability in regional disparity analyses.

## Statistical analysis

In accordance with the NDB Open Data disclosure rules, procedure counts of fewer than 10 cases were not reported and were treated as “no cases” for analysis.

For each surgical category, we calculated the maximum and minimum numbers of procedures per 100,000 population and per board-certified ophthalmologist, and also determined the prefectural disparities. Associations between surgical volume and population size or number of ophthalmologists were evaluated using Pearson’s correlation coefficient, and P < 0.05 indicated statistical significance.

Regional disparities were quantified using Gini coefficients, and coefficients of variation were also calculated as complementary measures of variability.

In addition to population-based estimates, age-adjusted expected surgical cases were calculated to account for differences in prefectural age structure, and Gini coefficients were computed based on these adjusted values. The mean age of each prefecture in 2022 was obtained from population estimates published by the Statistics Bureau of the Ministry of Internal Affairs and Communications, Japan, and adjustment was performed using the ratio of each prefecture’s mean age to the national average. Statistical analyses were performed using R (version 4.5.2). Gini coefficients below 0.1 generally indicate low inequality, whereas values exceeding 0.3 suggest substantial regional disparity. Because the NDB Open Data suppresses cell counts fewer than 10, suppressed values were treated as zero in the primary analysis. Sensitivity analyses were performed by imputing suppressed cells as 5 and 9 cases to examine the robustness of inequality and correlation estimates. This study is reported in accordance with the Strengthening the Reporting of Observational Studies in Epidemiology (STROBE) guidelines. A completed STROBE checklist is provided as Supporting Information.

## Results

### Surgical volumes and distribution of ophthalmologists

In the fiscal year 2022, 1,777,502 cataract surgeries, 154,336 vitrectomies, 80,753 glaucoma surgeries, and 2,895 corneal transplantations were performed nationwide. The overall and subgroup-specific surgical volumes for each procedure, together with the maximum and minimum values across prefectures, are summarized in Table 2. The proportion of inpatient procedures among all surgeries varied substantially by disease; and cataract surgeries, vitrectomies, glaucoma surgeries, and corneal transplantation accounted for 32.3%, 68.5%, 58.9%, and 93.6%, respectively, of the total number of procedures. The mean number of board-certified ophthalmologists per prefecture was 257 and ranged from 52 to 1,970. The maximum and minimum values, max–min ratios, and Gini coefficients of the prefecture-level surgical volumes per 100,000 population for cataract surgery, vitrectomy, glaucoma surgery, and corneal transplantation are presented in Table 3. The ophthalmologist density per 100,000 population is also shown.

**Table 2:** Total and prefecture-level surgical volumes and number of ophthalmologists in Japan (FY2022).

Surgical procedure	Classification	Total number of claims	Maximum value by prefecture	Minimum value by prefecture
**Cataract surgery**	**All**	**1,777,502**	**174,578**	**8,500**
**Vitreoretinal surgery**	**All**	**154,336**	**16,883**	**600**
	**Group 1**	**12,551**	**1,329**	**35**
	**Group 2**	**135,338**	**14,583**	**556**
	**Group 3**	**6,447**	**971**	**0**
**Glaucoma surgery**	**All**	**80,753**	**7,239**	**235**
	**Group 1**	**21,841**	**2,408**	**57**
	**Group 2**	**58,912**	**4,831**	**103**
**Corneal transplantation**	**All**	**2,895**	**393**	**0**
Board-certified ophthalmologists*		**12,080**	**1,970**	**52**

**Table 3:** Prefecture-level surgical volumes per 100,000 population and regional disparity indices.

Procedure	Classification	Mean number	Maximum number	Minimum number	Ratio	Gini coefficient	Age-adjusted Gini coefficient
Cataract surgery	All	1481.9	1886.2	1072.8	1.8	0.064	0.065
Vitreoretinal surgery	All	126.8	213.6	65.7	3.3	0.125	0.120
	Group 1	10.7	22.1	2.4	9.2	0.199	
	Group 2	110.5	196.0	54.1	3.6	0.106	
	Group 3	5.6	18.7	0.0	NA	0.305	
Glaucoma surgery	All	70.5	190.0	25.3	7.5	0.190	0.257
	Group 1	19.0	54.4	4.8	11.3	0.228	
	Group 2	51.5	142.5	11.1	12.9	0.186	
Corneal transplantation	All	2.0	5.8	0.0	NA	0.351	0.453
Board-certified ophthalmologists*		9.0	14.0	5.7	2.5	0.135	

The max–min ratios and Gini coefficients were 1.8 and 0.064 for cataract surgery, 3.3 and 0.125 for vitrectomy, and 7.8 and 0.190 for glaucoma surgery, respectively. For corneal transplantation, the max–min ratio could not be calculated because several prefectures reported zero cases, and the Gini coefficient was 0.351.

The coefficients of variation showed a similar pattern, with the lowest variability observed for cataract surgery and the highest for corneal surgery, supporting the robustness of the observed regional disparities.

Gini coefficients calculated using age-adjusted expected values showed consistent trends, with the highest inequality observed for corneal surgery and the lowest for cataract surgery. These findings indicate that the observed disparities are not solely explained by differences in age structure across prefectures.

**Fig 1:**
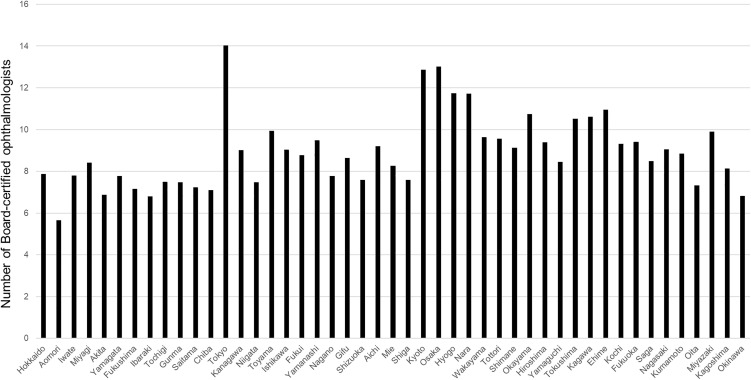
Distribution of ophthalmologist density across prefectures in Japan. Number of board-certified ophthalmologists per 100,000 population by prefecture.

**Fig 2:**
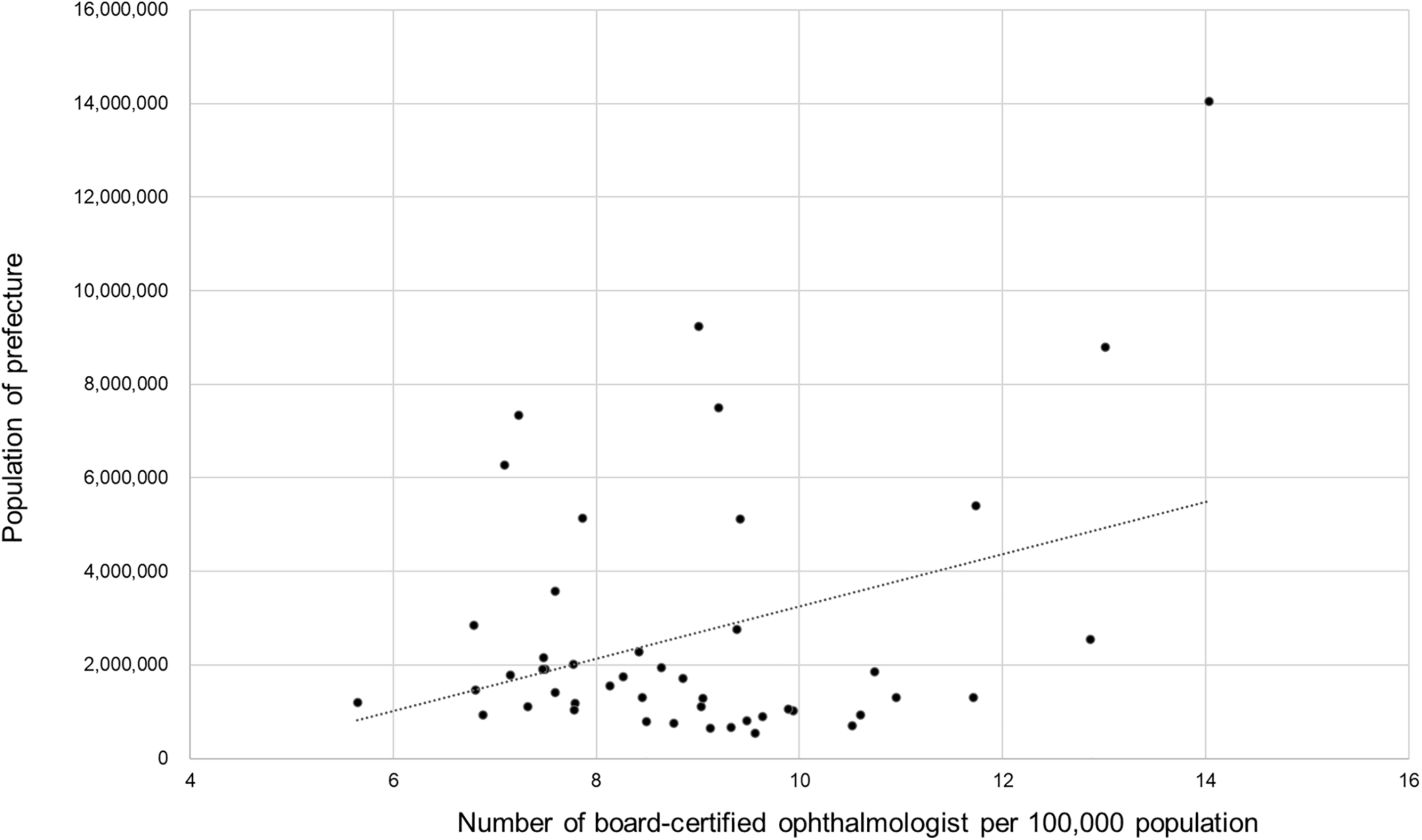
Association between prefectural population size and ophthalmologist density. A significant positive correlation was observed between population size and ophthalmologist density (r = 0.352, p = 0.015).

Ophthalmologist density per 100,000 population ranged from 14.0 in Tokyo to 5.7 in Aomori Prefecture, representing a 2.5-fold difference, with a Gini coefficient of 0.135. According to the Gini coefficients, regional disparities were smallest for cataract surgery, mild for vitrectomy, moderate for glaucoma surgery, and most pronounced for corneal transplantation. The ophthalmologist density per 100,000 population by prefecture is shown in Fig 1. Ophthalmologist density was positively correlated with prefectural population size (Fig 2; r = 0.352, p = 0.015).

**Fig 3:**
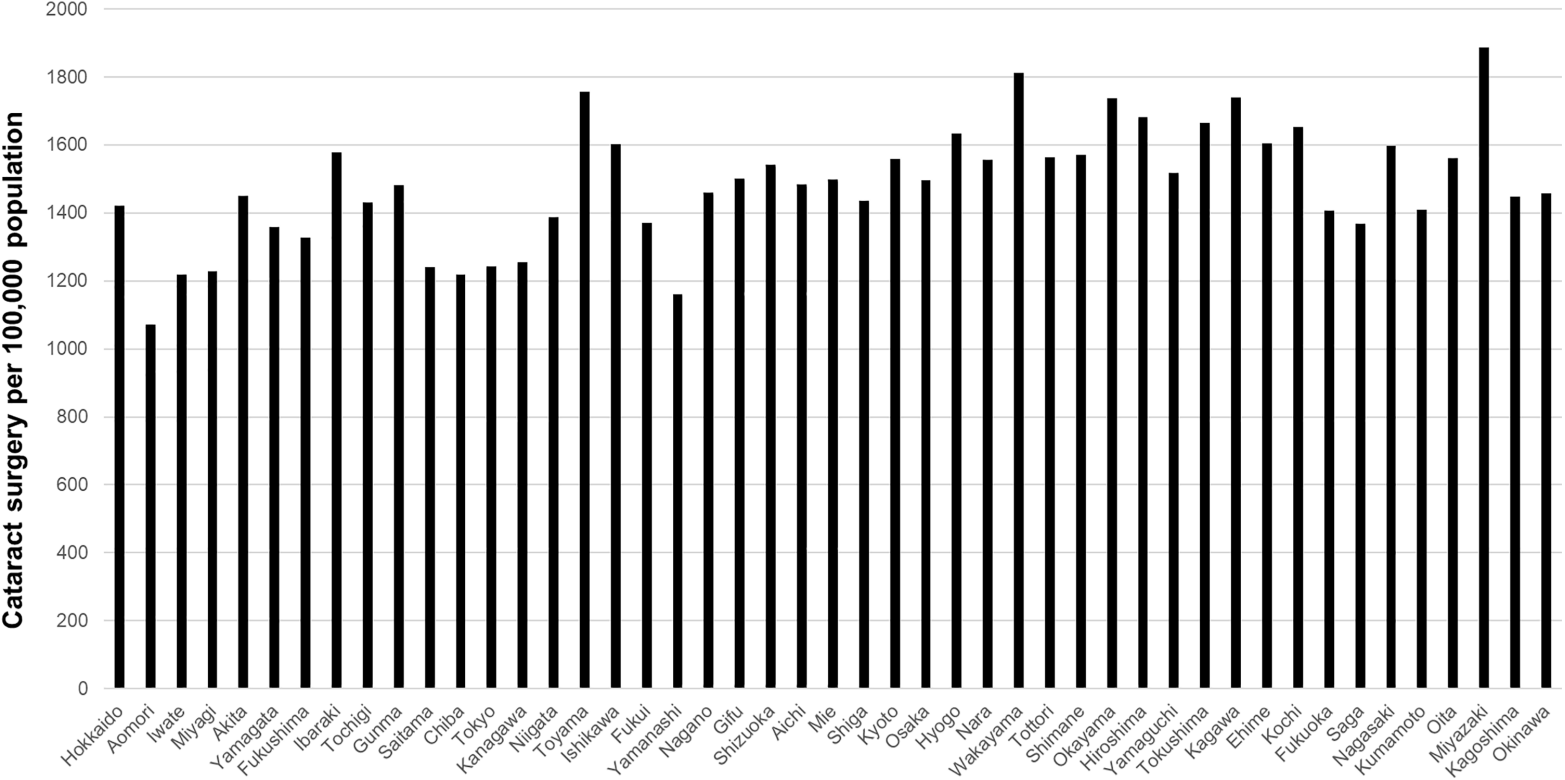
Prefecture-level cataract surgical volume per 100,000 population. Regional variation was relatively small.

**Fig 4:**
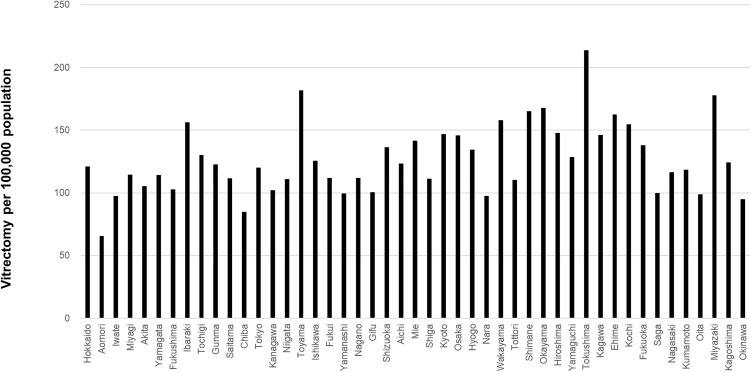
Prefecture-level vitrectomy surgical volume per 100,000 population. Regional variation was relatively small.

**Fig 5:**
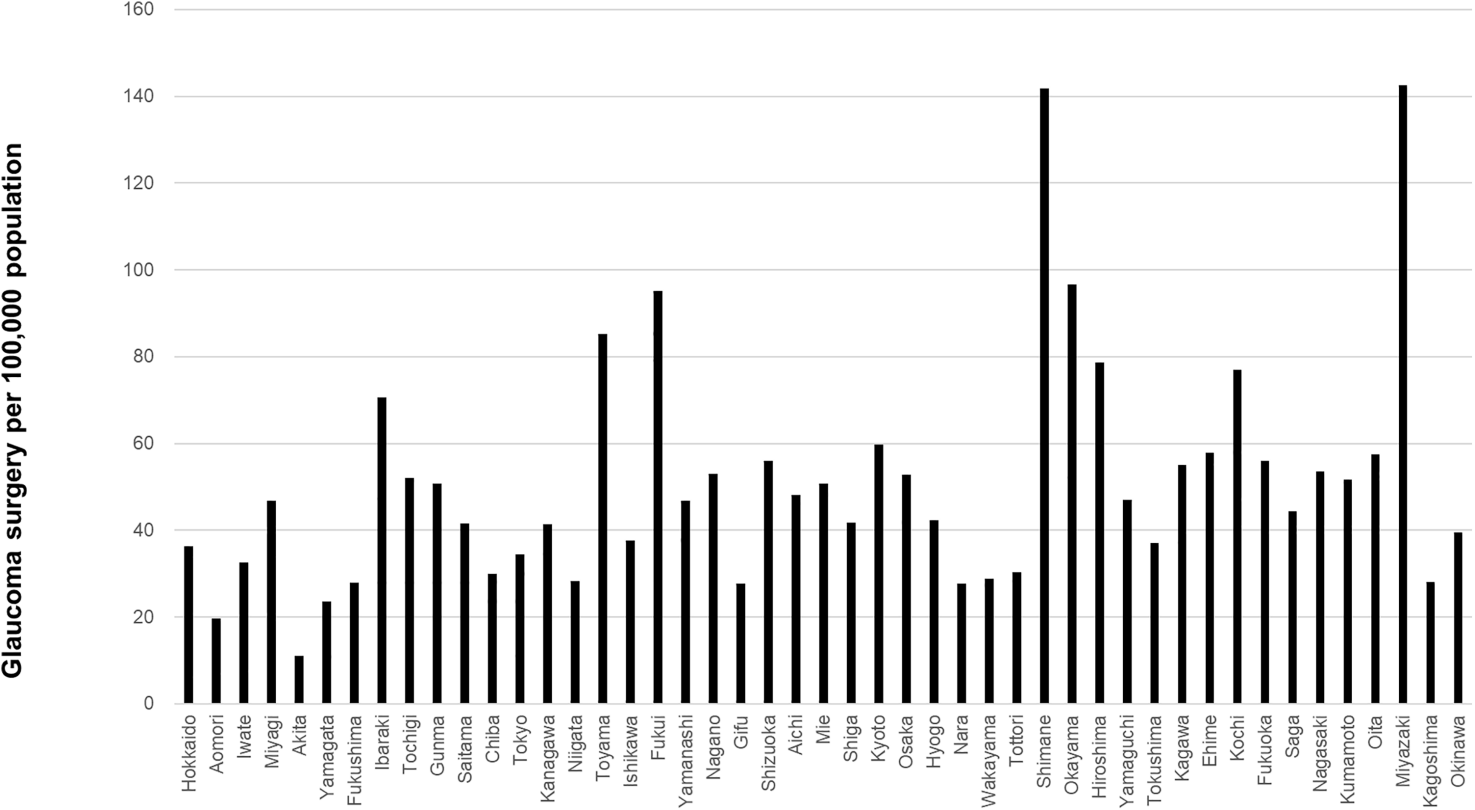
Prefecture-level glaucoma surgical volume per 100,000 population. Greater regional disparities were observed.

**Fig 6:**
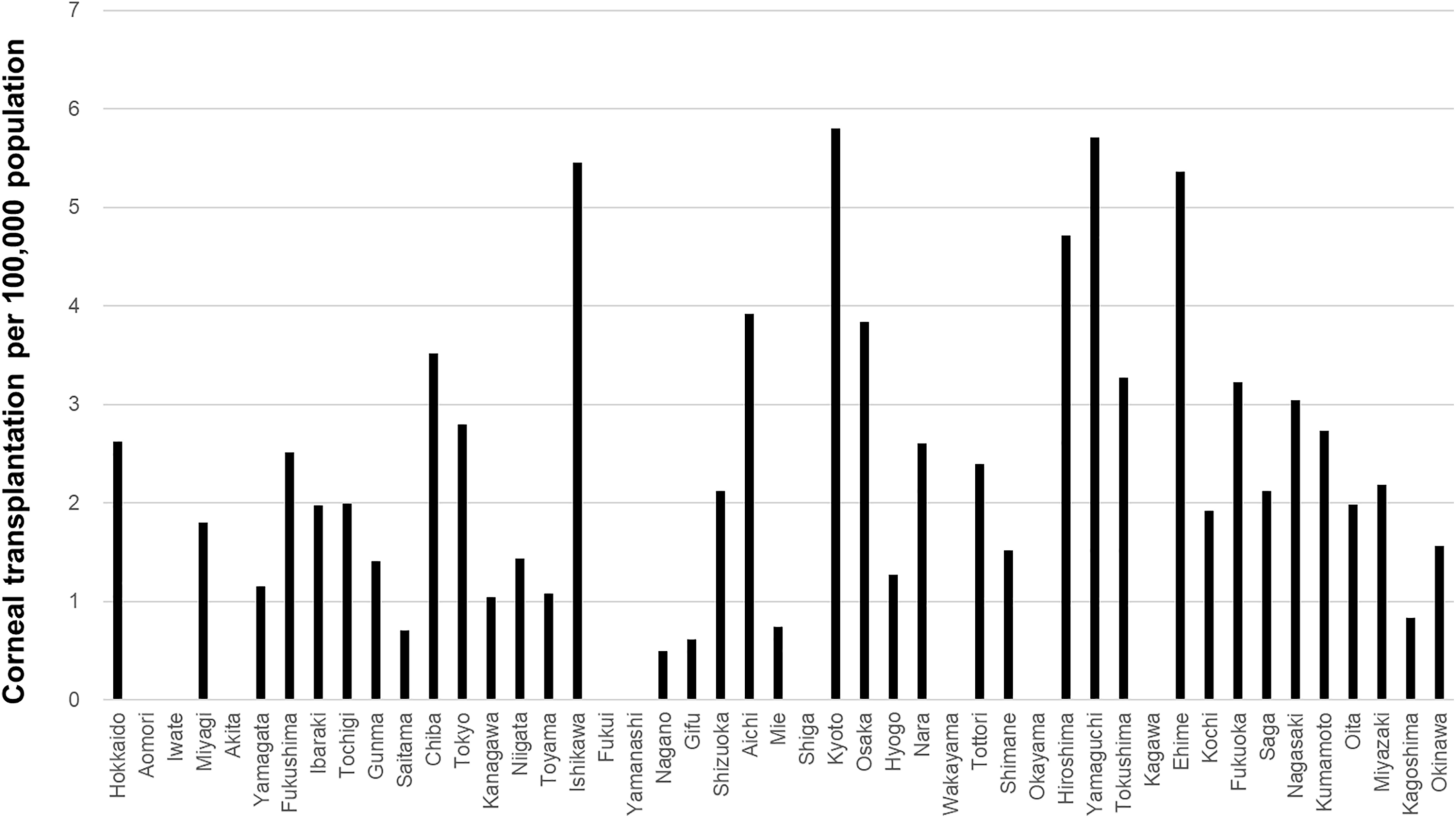
Prefecture-level corneal transplantation volume per 100,000 population. Greater regional disparities were observed, and several prefectures reported no cases.

The prefecture-level surgical volumes per 100,000 population for cataract surgery, vitrectomy, glaucoma surgery, and corneal transplantation are shown in Figs 3–6, respectively. Regional variation was relatively small for cataract surgery and vitrectomy, whereas greater disparities were observed for glaucoma surgery, and several prefectures reported no corneal transplantation under the current protocol.

### Group-based comparison of vitrectomy and glaucoma surgery per 100,000 population

With respect to vitrectomy, the mean number of surgeries per 100,000 population differed substantially among the three groups, with 10.7 surgeries in Group 1, 110.5 surgeries in Group 2, and 5.6 surgeries in Group 3. The corresponding Gini coefficients were 0.125, 0.199, and 0.305, respectively, indicating that Group 3, which comprises more technically demanding procedures, exhibited greater regional disparity than did the other groups.

For glaucoma surgery, the mean number of procedures per 100,000 population was 19.0 cases in Group 1 and 51.5 cases in Group 2, which demonstrates a marked difference between groups. The Gini coefficients were 0.228 for Group 1 and 0.188 for Group 2, indicating moderate regional disparities in both groups. Sensitivity analyses using alternative imputations for suppressed cells (5 or 9 cases) yielded similar Gini coefficients and correlation estimates, indicating that the results were robust to the treatment of suppressed values.

### Association between surgical volume and ophthalmologist density per 100,000 population

**Fig 7:**
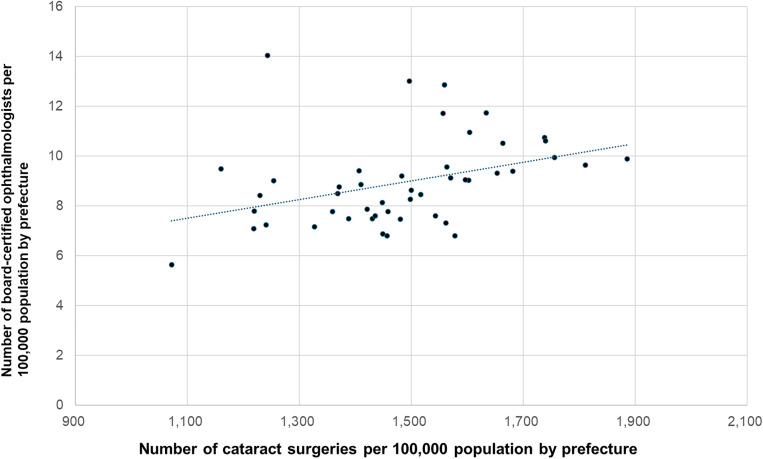
Association between cataract surgical volume and ophthalmologist density. A significant positive correlation was observed.

**Fig 8:**
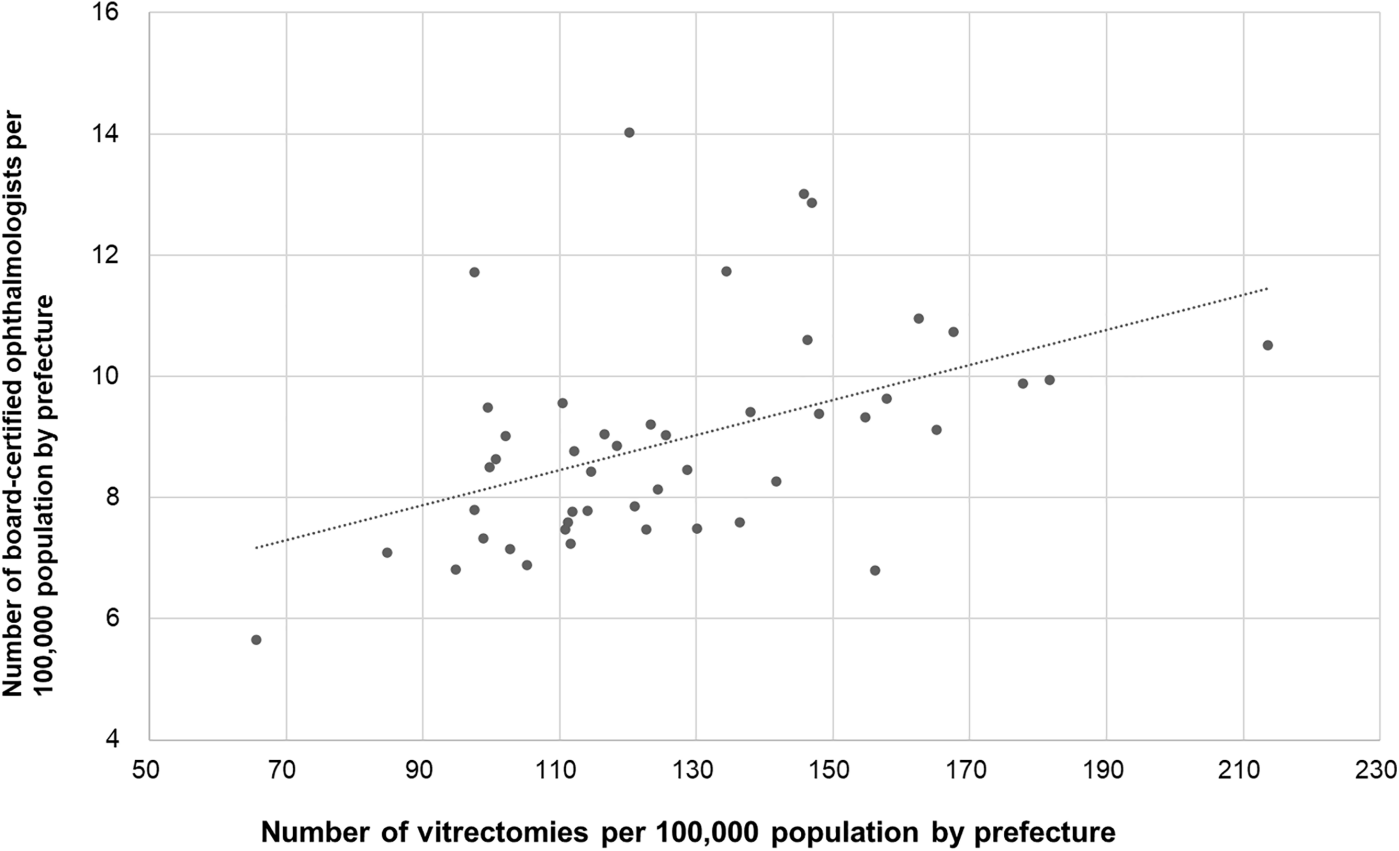
Association between vitrectomy surgical volume and ophthalmologist density. A significant positive correlation was observed.

We examined the associations between ophthalmologist density per 100,000 population and surgical volume per 100,000 population for cataract surgery, vitrectomy, glaucoma surgery, and corneal transplantation. Cataract surgery was significantly positively correlated with ophthalmologist density (r = 0.374, p = 0.0097; Fig 7). Similarly, vitrectomy was significantly positively correlated (r = 0.465; p < 0.001; Fig 8) with ophthalmologist density, as was corneal transplantation (r = 0.353; p = 0.0148; Fig 10).

**Fig 9:**
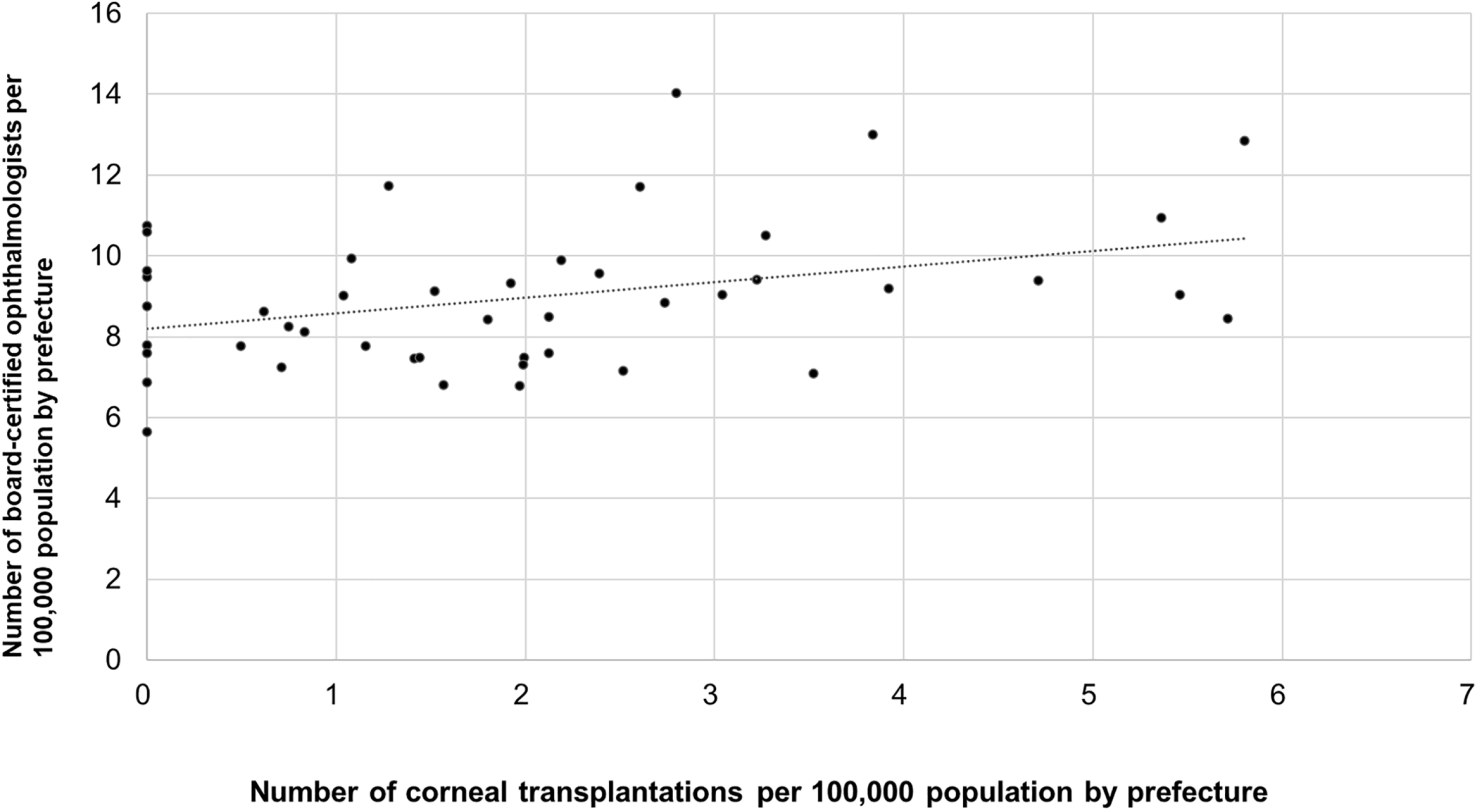
Association between glaucoma surgical volume and ophthalmologist density. No significant association was observed.

In contrast, the association between glaucoma surgery and ophthalmologist density did not reach statistical significance (r = 0.212, p = 0.15; Fig 9).

### Association between surgeries per ophthalmologist and ophthalmologist density per 100,000 population

We next examined the associations among ophthalmologist density per 100,000 population, an index of workforce availability, and the number of surgeries performed per ophthalmologist for each procedure. A significant inverse correlation was observed for cataract surgery (r = −0.774, p < 0.0001; Fig 11) and vitrectomy (r = −0.347, p = 0.017; Fig 12), which indicates a greater per-physician surgical burden in regions with lower ophthalmologist density. In contrast, no significant associations were observed for glaucoma surgery (r = −0.082; Fig 13) or corneal transplantation (r = 0.12; Fig 14.

**Fig 10:**
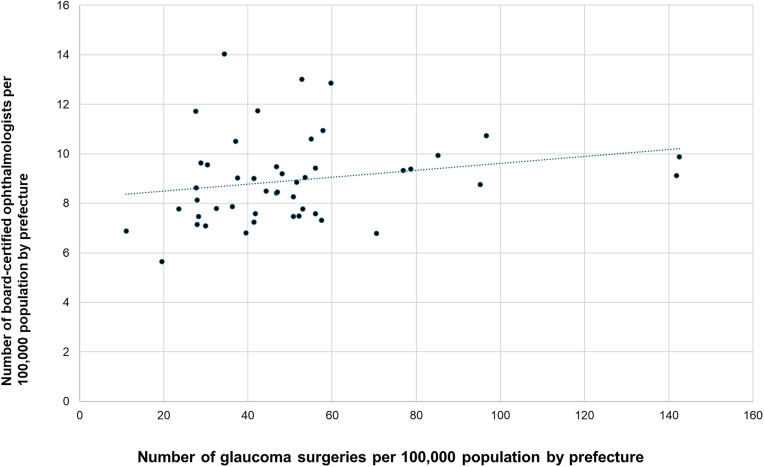
Association between corneal transplantation volume and ophthalmologist density. A significant positive correlation was observed.

**Fig 11:**
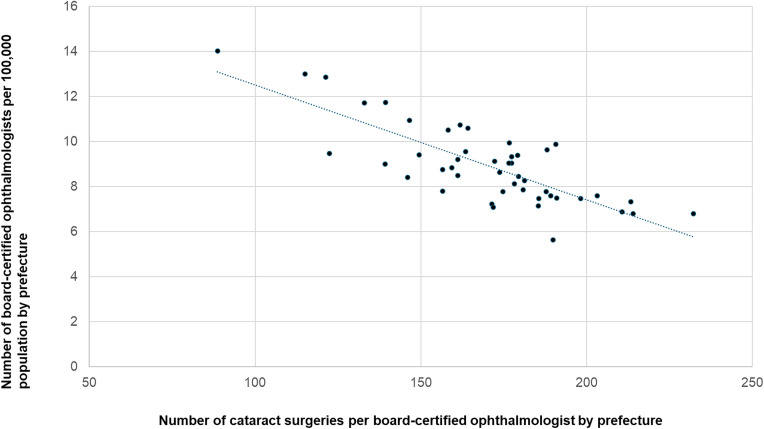
Association between cataract surgeries per ophthalmologist and ophthalmologist density. A significant inverse correlation was observed.

**Fig 12:**
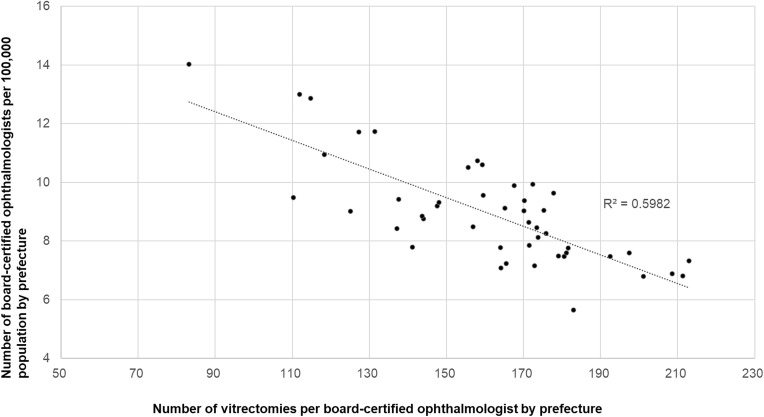
Association between vitrectomies per ophthalmologist and ophthalmologist density. A significant inverse correlation was observed.

**Fig 13:**
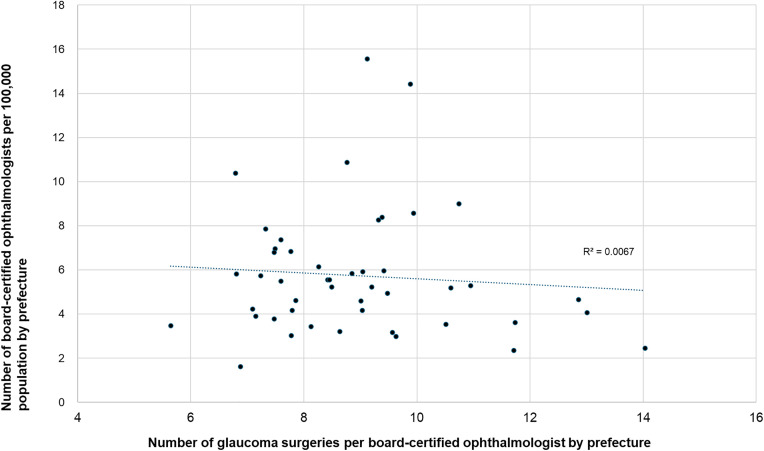
Association between glaucoma surgeries per ophthalmologist and ophthalmologist density. No significant association was observed.

**Fig 14:**
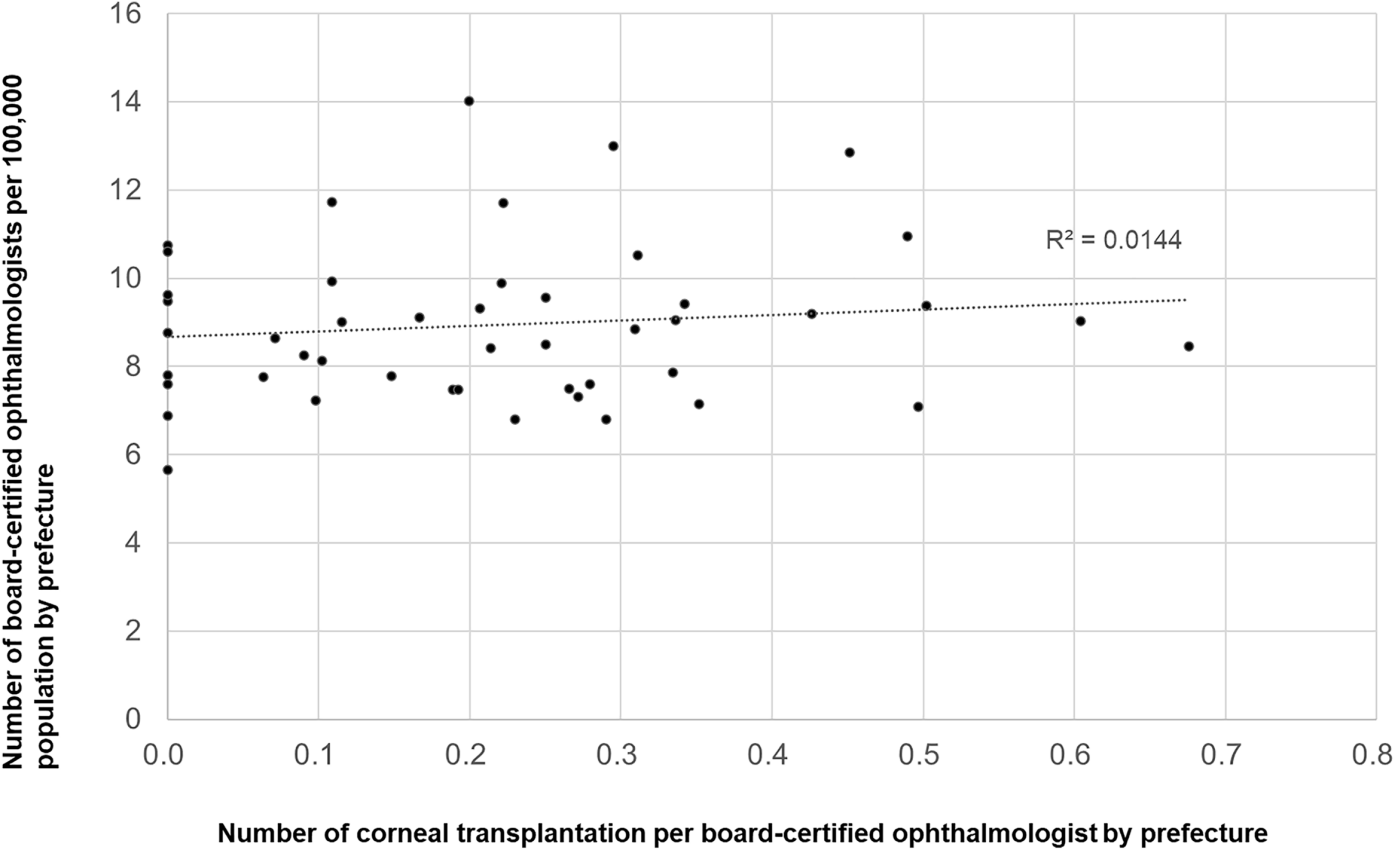
Association between corneal transplantations per ophthalmologist and ophthalmologist density. No significant association was observed.

## Discussion

### Summary

In this study, we investigated the current status of major ophthalmic surgeries performed in Japan, regional disparities in surgical volumes, and their associations with the distribution of board-certified ophthalmologists. We found that cataract surgery, which accounted for the greatest surgical volume, exhibited minimal regional disparity. In contrast, vitrectomy and glaucoma surgery showed overall mild regional disparities, with a tendency toward greater disparity for more technically demanding procedures. Corneal transplantation demonstrated moderate to substantial regional disparity. Collectively, these findings indicate the presence of regional inequalities in the provision of ophthalmic surgery in Japan. Importantly, these patterns remained consistent even after adjusting for regional differences in age structure, suggesting that the observed disparities reflect underlying differences in healthcare delivery rather than demographic variation alone.

Cataract surgery is the most common and central procedure in ophthalmology and is prioritized in surgical training programs for ophthalmologists. Consequently, a large proportion of ophthalmologists who perform surgery are trained to perform cataract surgery. In addition, cataract surgeries constitute a high proportion of outpatient cases and are frequently performed in clinics without inpatient beds. These factors likely contribute to the relatively small regional variation observed for cataract surgery.

In contrast, vitrectomy, glaucoma surgery, and corneal transplantation require a higher level of surgical expertise, and surgical training for these procedures requires substantial time and effort. Furthermore, the proportion of outpatient cases is lower, and these procedures are more commonly performed in institutions with inpatient facilities, which limits the number of facilities capable of providing such surgeries. These factors are likely to contribute to the greater regional disparities observed for these specialized ophthalmic procedures.

When the procedural groups were analyzed, regional disparities were found to be more pronounced for higher-complexity vitrectomy and glaucoma surgeries. In recent years, less invasive approaches to vitrectomy and glaucoma surgery have become increasingly widespread. In particular, the rapid adoption of minimally invasive glaucoma surgery (MIGS), which imposes a lower burden on both surgeons and patients, has been reported in glaucoma management [1]. In contrast, conventional filtering surgery requires complex intraoperative techniques and intensive postoperative management, which may limit the expansion of the surgeon workforce capable of performing these procedures. Such circumstances may also contribute to the widening regional disparities observed in glaucoma surgery.

The substantial regional disparity observed in corneal transplantation may be partly attributable to an insufficient number of qualified surgeons. In addition, the availability of donor corneas may play an important role. Most institutions in Japan rely on domestic eye banks for transplantable corneas; however, an insufficient supply of donor tissue may limit the number of procedures performed. Although some institutions use corneas obtained from overseas eye banks, differences in the availability and organization of donor cornea procurement systems may further contribute to regional variation. In the present analysis, several prefectures reported no cases of corneal transplantation. Because the NDB Open Data suppresses results when the number of cases for a given item is fewer than 10, these prefectures were considered to have zero cases in our analysis, which may also have influenced the observed magnitude of regional disparity.

### Physician maldistribution: Ophthalmology in the context of other medical specialties

In Japan, the total number of physicians has increased in parallel with the expansion of medical school enrollment; however, physician maldistribution has emerged as a major concern in recent years [6,7]. Surveys conducted by the Ministry of Health, Labour and Welfare have reported an approximately twofold difference in physician density between Tokyo, which has the greatest number of physicians, and prefectures, which have the lowest physician supply [14]. Although prefectural disparities in physician numbers have been widely recognized, our analysis further demonstrated that ophthalmologist density per 100,000 population was markedly higher in Tokyo, with a 2.5-fold regional difference observed nationwide. Ophthalmologist density tended to be lower in eastern Japan, particularly in northern regions, than in western Japan.

The number of board-certified ophthalmologists is closely related to the provision of ophthalmic care. Consistent with this, prefectures with fewer ophthalmologists—primarily in northern and rural regions—tended to have lower surgical volumes. Because surgical volume is correlated with ophthalmologist density, physician maldistribution may contribute to regional disparities in ophthalmic surgical care. Therefore, reducing regional disparities in the distribution of ophthalmologists may be important to ensure equitable access to appropriate ophthalmic services.

In the present analysis, surgical volumes tended to be greater in regions with a greater number of ophthalmologists per 100,000 population, which were predominantly urban areas. Accordingly, ophthalmic surgeries were more frequently performed in urban regions where ophthalmologists were concentrated. Ophthalmologist density was inversely correlated with the number of surgeries performed per ophthalmologist for cataract surgery and vitrectomy. In contrast, no such association was observed for glaucoma surgery or corneal transplantation. The lack of correlation between ophthalmologist density and glaucoma surgery volume may reflect the unique characteristics of glaucoma management. Unlike cataract surgery, which is widely performed across institutions, glaucoma surgery tends to be concentrated in specialized centers with expertise in surgical management. In addition, regional variations in treatment strategies—such as the preference for medical therapy over surgical intervention—and differences in disease detection and referral patterns may also contribute to this finding. These findings may indicate that demand-driven, high-volume procedures in densely populated urban areas are relatively well supplied.

With respect to less frequently performed procedures, such as glaucoma surgery and corneal transplantation, certain prefectures exhibited disproportionately high surgical volumes regardless of population size. This pattern may reflect the presence of surgeons or institutions specializing in these procedures, independent of regional population density. While such specialization may enable the provision of advanced ophthalmic care despite limited local resources, performing a high volume of surgeries with limited workforce capacity may increase the burden on healthcare providers. Moreover, in regions with relatively low ophthalmologist density, a concentration of specific procedures may be associated with greater variability in surgical approaches. In areas with limited medical resources, it is therefore important to establish systems that can address a broad range of patient needs. In addition, regions that serve as surgical hubs may require structured frameworks to ensure the continuity and transfer of surgical expertise.

### The need for and challenges of centralizing ophthalmic care

In Japan, the proportion of elderly households—particularly households consisting of elderly couples or individuals living alone—has increased rapidly in recent years. According to a report by the Ministry of Health, Labour and Welfare, households headed by individuals aged 65 years or older accounted for approximately 31.4% of all households in 2024, while single-person elderly households accounted for approximately 16% [15]. These demographic changes pose substantial challenges to the delivery of appropriate care, including early disease detection, outpatient visits, and access to surgical treatment.

From the perspective of future ophthalmic surgical care, a certain degree of centralization may be necessary for procedures with relatively low surgical volumes. However, such centralization must be carefully balanced against patient access to medical institutions, particularly for older adults with limited mobility. At present, the ophthalmic care system in Japan does not seem to have adequately addressed these challenges. Therefore, the establishment of an equitable and high-quality ophthalmic care delivery system that accounts for both procedural specialization and patient accessibility is an urgent priority.

### Comparison with global evidence

Previous studies that have examined regional disparities in cataract surgery have reported higher Gini coefficients than those reported in the present study, which indicates that regional disparity in cataract surgical provision is a global concern [16–18]. This tendency is particularly pronounced in regions with less developed social and healthcare infrastructures. Although direct comparisons across countries are limited by differences in healthcare systems and study methodologies, regional disparities in cataract surgery may be relatively small in Japan compared with those in many other countries. In the United States, for example, disparities in the availability of rural subspecialist surgeons have been reported [9].

Studies that have comprehensively evaluated regional disparities across a broad range of ophthalmic surgeries, such as those investigated in the present analysis, remain scarce. The prevalence of glaucoma is expected to increase worldwide in the coming decades [19], which may lead to a corresponding increase in the number of patients who require surgical intervention. While prior studies have examined access to glaucoma care [20] and socioeconomic disparities in glaucoma management [21], few investigations have focused on regional variation in glaucoma surgical volume.

Similarly, global data on corneal transplantation remain limited. Gain et al. reported that approximately 50% of the global population lacks access to corneal transplantation and that nearly 80% of corneal transplantations are performed in a small number of countries [22]. However, detailed analyses of regional disparities within individual countries are scarce. With respect to vitrectomy, Wubben et al. described trends in vitrectomy application and reported that procedure rates varied by region, age, sex, and race [23]. Montazeri et al. reported temporal trends and regional differences in surgical techniques for vitrectomy in the United States but did not specifically address regional disparities in surgical volume [24]. Moreover, few studies have investigated the relationship between vitrectomy volume and the distribution of specialized medical resources, such as board-certified ophthalmologists.

Overall, global evidence regarding regional disparities is limited. Given that Japan’s healthcare system is generally considered well developed in a global context, regional disparities in these procedures may be even more pronounced in other countries.

### Limitations

This study has several limitations. First, our analysis was based on the NDB Open Data, which aggregates nationwide health insurance claims and specific health checkup data and covers approximately 99% of all medical claims in Japan. This extensive coverage enables a comprehensive assessment of surgical practice nationwide. However, the database is structured according to clinical procedure codes, which limits the ability to capture detailed information on the increasingly diverse range of surgical techniques currently in use.

Second, although we used the most recent publicly available NDB Open Data from the fiscal year 2022, surgical practices—particularly those in glaucoma—have evolved rapidly in recent years. Minimally invasive gynecologic surgery (MIGS) has expanded substantially, as the PRESERFLO MicroShunt was approved for reimbursement in 2022, while the Hydrus Microstent was approved in 2024, which was followed by rapid adoption [1–4]. In the present dataset, some prefectures reported no outflow procedures; however, surgical volumes may have changed with the increasing adoption of MIGS. Future studies using more recent data will be necessary to evaluate temporal trends in surgical volume.

Third, in corneal transplantation, partial-thickness procedures such as endothelial keratoplasty have increased, resulting in a declining proportion of full-thickness penetrating keratoplasty [25]. Similarly, advances in surgical techniques have been introduced for vitrectomy. Because detailed, procedure-specific subclassifications were not available in the present dataset, we were unable to analyze these surgical subtypes separately, which represents an important area for future investigation. In addition, annual procedure counts fewer than 10 cases were not disclosed in the dataset and were treated as zero in the present analysis. This approach may have led to underestimation of surgical volumes and could have influenced inequality measures, particularly for low-frequency procedures such as corneal transplantation. Therefore, the findings for rare procedures should be interpreted with caution.

Fourth, although surgical volume data were derived from the 2022 NDB Open Data, ophthalmologist workforce data and population statistics were obtained from 2025 and 2024 sources, respectively. While these data do not correspond to the same year, major changes in ophthalmologist distribution and population demographics over this period are unlikely; therefore, the impact on our results is expected to be limited.

Fifth, because the NDB data are based on the location of surgical procedures rather than patients’ residence, patient mobility across prefectures may have influenced the observed regional distribution. In particular, complex procedures such as corneal transplantation and advanced vitreoretinal surgeries are often performed in high-volume tertiary centers located in urban areas. This may lead to an overestimation of regional inequality, as reflected by higher Gini coefficients. Finally, although our analysis focused primarily on the association between surgical volume and ophthalmologist density, multiple additional factors, including socioeconomic status, educational level, age distribution, disease prevalence, and economic conditions, may influence surgical utilization. Moreover, the provision of surgical care depends not only on physicians but also on allied health professionals such as nurses and other medical staff. The incorporation of these factors into future analyses will be important for a more comprehensive understanding of regional disparities in ophthalmic surgery. This ecological analysis at the prefectural level does not allow inference at the individual patient or physician level.

### Conclusion

In an aging society, the number of patients with ophthalmic diseases is increasing, and corresponding increases in the volume and complexity of ophthalmic surgical care are anticipated. Ophthalmic practice has become increasingly advanced and specialized and thus requires greater acquisition of technical skills and knowledge than in the past. As a result, disparities in the availability of surgical procedures may increase, and the burden on healthcare providers may also increase. These trends raise concerns about further inequities in access to appropriate ophthalmic care. The development of strategies that ensure equitable access to high-quality ophthalmic services and that prevent excessive burden on healthcare providers will be increasingly important in the future.

## Supporting information

S1 File STROBE. (DOCX)
