# Supplementary material for: Disparity of ophthalmic surgeries in Japan
Source: PLoS One. 2026 Apr 24;21(4):e0347587. doi: 10.1371/journal.pone.0347587 (PMC13108813; doi:10.1371/journal.pone.0347587)
Supplement: S1 File — (DOCX) [file pone.0347587.s001.docx]

STROBE Statement—Checklist of items that should be included in reports of cross-sectional studies (Journal-optimized version)

| Section | Item No. | Recommendation | Manuscript Description | Location (Page/Line) |
| --- | --- | --- | --- | --- |
| Title/Abstract | 1a | Study design indicated | Cross-sectional analysis of nationwide claims data | Abstract |
| Title/Abstract | 1b | Structured summary | Purpose/Methods/Results/Conclusions provided | Abstract |
| Introduction | 2 | Background | Aging population and disparity described | Introduction |
| Introduction | 3 | Objectives | Prefecture-level disparities evaluated | Introduction |
| Methods | 4 | Study design | Cross-sectional study stated | Methods |
| Methods | 5 | Setting | Japan, FY2022, NDB Open Data | Methods |
| Methods | 6 | Participants | Nationwide aggregated dataset | Methods |
| Methods | 7 | Variables | Surgical volumes, density, Gini | Methods |
| Methods | 8 | Data sources | NDB, population, society data | Methods |
| Methods | 9 | Bias | A400, suppression, migration discussed | Methods/Discussion |
| Methods | 10 | Study size | Nationwide dataset (~99%) | Methods |
| Methods | 11 | Quantitative variables | Rates, Gini, ratios | Methods |
| Methods | 12 | Statistical methods | Correlation, Gini, sensitivity analysis | Methods |
| Results | 13 | Participants | Not applicable | — |
| Results | 14 | Descriptive data | Tables 2–3 | Results |
| Results | 15 | Outcome data | Surgical volumes | Results |
| Results | 16 | Main results | Gini, correlations | Results |
| Results | 17 | Other analyses | Subgroup & sensitivity | Results |
| Discussion | 18 | Key results | Complexity vs disparity | Discussion |
| Discussion | 19 | Limitations | Fully described | Discussion |
| Discussion | 20 | Interpretation | Healthcare implications | Discussion |
| Discussion | 21 | Generalisability | Global context discussed | Discussion |
| Other | 22 | Funding | None | Statement |
| Other | 23 | Ethics | Not required (anonymous data) | Methods |
